# The common promoter polymorphism rs11666254 downregulates *FPR2*/*ALX* expression and increases risk of sepsis in patients with severe trauma

**DOI:** 10.1186/s13054-017-1757-3

**Published:** 2017-07-06

**Authors:** Han Zhang, Yao Lu, Guixiang Sun, Fang Teng, Nian Luo, Jianxin Jiang, Aiqing Wen

**Affiliations:** Department of Blood Transfusion, Research Institute of Surgery, Daping Hospital, Third Military Medical University, No. 10 Changjiang Branch Road, Daping District, Chongqing, 400042 China

**Keywords:** FPR2/ALX, Promoter polymorphism, Sepsis, Severe trauma

## Abstract

**Background:**

Formyl peptide receptor 2-lipoxin receptor (FPR2/ALX) modulates the anti-inflammatory response and therefore may be a target for treating sepsis. The purpose of this study was to investigate the association between genetic variants of the *FPR2/ALX* gene and sepsis after severe trauma as well as to further analyze the functions of sepsis-related genetic polymorphisms.

**Methods:**

Three tag single-nucleotide polymorphisms (tag SNPs) that captured all common alleles across the *FPR2/ALX* genomic region were genotyped using pyrosequencing in an initial sample consisting of 275 patients with severe trauma. The rs11666254 polymorphism, which had statistical significance, was genotyped in an additional 371 patients, and logistic regression analysis was performed to determine associations between the *FPR2/ALX* gene polymorphism and sepsis susceptibility after severe trauma. The messenger RNA (mRNA) and protein levels of FPR2/ALX in the lipopolysaccharide-stimulated white blood cells of trauma patients were determined by performing quantitative polymerase chain reactions and Western blot analysis. Tumor necrosis factor (TNF)-α production was measured by enzyme-linked immunosorbent assay. The effects of the promoter polymorphism rs11666254 on the transcription activity of *FPR2/ALX* were analyzed using a luciferase reporter assay.

**Results:**

Among the three tag SNPs, only the rs11666254 polymorphism was found to be significantly associated with sepsis in trauma patients, and this association persisted after a pooled analysis of all 646 trauma patients, which showed that patients who carried the A allele of rs11666254 had a significantly higher risk of developing sepsis than individuals who carried the G allele. This SNP was also significantly associated with lower FPR2/ALX mRNA and protein expression as well as higher TNF-α production from the peripheral blood leukocyte response to bacterial lipoprotein stimulation. In addition, the rs11666254 polymorphism could significantly decrease the promoter activity of the *FPR2/ALX* gene.

**Conclusions:**

The rs11666254 polymorphism in the *FPR2/ALX* gene is a functional SNP that increases sepsis susceptibility in patients after traumatic injury.

**Electronic supplementary material:**

The online version of this article (doi:10.1186/s13054-017-1757-3) contains supplementary material, which is available to authorized users.

## Background

Trauma is a major and costly public health problem around the world, and it is the fourth leading cause of death among young adults in China [1, 2]. Infectious disease, sepsis, and multiple organ dysfunction syndrome (MODS) remain important complications for patients who survive major trauma [3, 4]. Therefore, preventing sepsis and MODS is crucial in the treatment of patients who survive major trauma.

A single-nucleotide polymorphism (SNP) is a variation in a single nucleotide that occurs at a specific position in the genome where each variation is present to some appreciable degree within a population. Increasing evidence suggests that SNPs are critical for determining interindividual differences in both inflammatory responses and clinical outcomes in sepsis patients [5, 6]. The previous results of our group and results reported by other groups have indicated that genetic variations within at least 38 genes from the proinflammatory signaling pathway, including pattern recognition receptors (*TLR1*, *TLR2*, *TLR4*, and *TLR9*), signal-transducing adaptor proteins (*cluster of differentiation 14 [CD14], LBP, MD2, HMG1*), and inflammatory cytokines of the immune system (*IL1A*, *IL1B*, *IL1RN*, *IL4*, *IL6*, *IL8*, *IL10*, *IL17F*, *IL18*, and *TNF-α*), are critical determinants of the magnitude of the immune inflammatory response, which profoundly affects the proinflammatory response to trauma and predisposes trauma patients to susceptibility or resistance to sepsis and MODS [7–14]. Therefore, identifying the polymorphisms and associated interindividual differences may assist with the risk stratification of trauma patients at the early stages of trauma and might contribute to developing new genetically based diagnostic and therapeutic interventions that alter host susceptibility to sepsis-related outcomes. However, these studies are not easily compared, owing to the relatively small sample sizes and different study populations [13]. Furthermore, the outcomes of these studies are sometimes different, which makes it more difficult to pool or compare results between these studies. Although some SNPs clearly appear to be associated with the disease, no definitive conclusions currently can be drawn regarding the influence of specific sequence variations on outcomes in trauma patients [13].

It has been demonstrated that an inappropriate immune inflammatory response contributes to the development of sepsis and MODS in major trauma patients [15, 16]. The development and resolution of inflammation may involve both proinflammatory and anti-inflammatory mechanisms [17, 18]. The resolution of inflammation is an active process that prevents damage to the host and is governed by specific proresolving mediators. Lipoxin A4 (LXA4) was a main endogenous stop signal of inflammation, and the responses were mediated by a specific G protein-coupled receptor called *formyl peptide receptor 2* (FPR2, which is also known as FPRL1 or ALXR). Human FPR2/ALX is highly expressed in myeloid cells and expressed at low levels in lymphocytes and dendritic, macrophage, and endothelial cells [17, 18]. FPR2/ALX conveys proresolving properties by provoking detachment of neutrophils, facilitating neutrophil apoptosis, and promoting macrophage efferocytosis and release of anti-inflammatory cytokines [19]. On the basis of these biological functions, activation of FPR2/ALX has been shown to be essential in models of experimental inflammation, such as sepsis [20–23], cerebral inflammation [24], acute lung injury [25, 26], and stroke [27]. Accumulating evidence shows that the FPR2/ALX expression level may have pathophysiological relevance. Additionally, the *Fpr2/3* gene (an orthologue to human *FPR2/ALX*) deficiency in mice impairs bacterial clearance and aggravates the host response in polymicrobial sepsis [28], which suggests that FPR2/ALX has a pivotal role in the development of sepsis. Simiele et al. have shown that a single-nucleotide mutation (A/G) located −220 bp upstream of the transcription start site reduces promoter activity by approximately 35% to 90% in vitro [29]. In addition, the messenger RNA (mRNA) and protein levels of FPR2/ALX are reduced by approximately ten- and threefold, respectively, in neutrophils of individuals carrying the A/G mutation compared with cells from individuals expressing the wild-type allele [29]. These findings suggest that genetic variants of *FPR2/ALX* may play an important role in regulating gene expression and ultimately influence the anti-inflammatory processes in patients with sepsis. However, there has not been any research on the clinical relevance of all the genetic variants of the *FPR2/ALX* gene with sepsis and MODS after major trauma.

In this study, to assess the comprehensive potential biological significance of all polymorphisms of the *FPR2/ALX* gene, a tag single-nucleotide polymorphism (tSNP) approach was used. The association of the tSNPs with the development of sepsis and MODS in patients with major trauma was evaluated.

## Methods

### Patients

The protocol for this study was approved by the ethical and protocol review committee of the Third Military Medical University, and informed consent was obtained from the participants or their next of kin (REB protocol number 41/2011). Patient confidentiality was preserved according to the guidelines for studies on human subjects. Trauma patients were recruited from the Department of Trauma Surgery of Daping Hospital and the Chongqing Emergency Medical Center, Chongqing, China, between 2005 and 2011. A total of 646 major trauma patients, comprising 523 men and 123 women, were recruited in the clinical relevance association study. After taking cost-effectiveness and higher efficiency considerations into account, the candidate tag SNPs were screened in a small sample population, then potential positive SNPs were validated in a larger sample population. Consequently, a cohort of 275 trauma patients was admitted between 1 January 2009 and 28 March 2011. Then, another cohort of 371 trauma patients was enrolled during the period between 1 January 2012 and 30 December 2015. All patients survived more than 48 h after admission and completed genotyping. The detailed inclusion and exclusion criteria have been described previously [14].

### Clinical evaluation

After admission, five factors were monitored in all participating major trauma patients, including respiratory conditions indicated by the ratio of partial pressure of arterial oxygen to the fraction of inspired oxygen, cardiovascular conditions indicated by the pressure-adjusted heart rate, renal conditions indicated by serum creatinine concentration, hematological conditions (platelet count), and hepatic conditions indicated by serum bilirubin concentration. Organ function was then scored using the methods described by Marshall et al. [30]. The assessment was based on the worst value indicators within 24 h during intensive care unit treatment. Patients were diagnosed with sepsis only if they met all of the following criteria: body temperature above 38.5 °C or below 36.5 °C, leukocyte count above 10 × 10^9^/L or below 4 × 10^9^/L, and clinical evidence of infection. Individuals who were blinded to the patients’ genotypes determined both their sepsis status and multiple organ dysfunction (MOD) scores.

### Selection of tag SNPs in *FPR2*/*ALX* gene

The GenBank database showed that the human *FPR2/ALX* gene was located on chromosome 19q13.3–13.4 [accession number NC_00019.9, chr19:52261453 to 52276779]. Three tag SNPs were selected to capture all common alleles at *r* ^2^ greater than 0.8 across the *FPR2/ALX* genomic region, including all exons, introns, the 5′-untranslated region (5′-UTR), the 3′-UTR, the 3-kb proximal promoter region, and the 3-kb downstream region. The three tag SNPs were rs11666254, rs17695052, and rs17695064. They were located on −1010A/G, 259A/G (exon 2, 3′-UTR), and 290C/T (exon 2, 3′-UTR), respectively. Tag SNPs were selected according to the HapMap Han Chinese in Beijing (CHB) data (version 3, release R2) using Haploview version 4.2 (update 24) (Additional file 1). The minimum minor allele frequency (MAF) for checking markers was set to 0.05. The most informative tag SNP was selected from each linkage disequilibrium block using the Tagger program in Haploview. To determine the possible functionality of the tag SNP (rs11666254) selected from the proximal promoter region of the *FPR2/ALX* gene, online software (http://www.genomatix.de/solutions/genomatix-software-suite.html, http://www.gene-regulation.com/, and http://www.genome.jp/tools/motif/) was used to analyze the effects of this SNP on potential transcription factor binding sites.

### Pyrosequencing

Tripotassium ethylenediaminetetraacetic acid-coated sterile tubes were used to store blood specimens collected from trauma patients. These samples were taken immediately upon admission so that blood transfusion could be performed as needed without affecting the results of the study. Genomic DNA was extracted from the whole blood of trauma patients using the Wizard Genomic DNA1 Purification Kit (Promega, Madison, WI, USA). The primers used to detect the *FPR2/ALX* SNPs were designed using Pyrosequencing^TM^ Assay Design Software [31] (Table 1). The detailed methods were described previously [14].

**Table 1:** Primers of the tag single-nucleotide polymorphisms of *FPR2/ALX*

SNP	Forward primer	Reverse primer	Length (bp)
rs11666254	Bio-CATGTTCCCTCCTCCGGATAT	GGGGCACGTAGTGATAGACAGA	107 bp
rs17695052	TTTTTGACTTCTGCCTATAC	Bio-AAAAACCTACAGCAAACATT	151 bp
rs17695064	TTTTTGACTTCTGCCTATAC	Bio-AAAAACCTACAGCAAACATT	151 bp

*SNP* Single-nucleotide polymorphism

### Ex vivo lipopolysaccharide stimulation of whole blood

A human whole-blood assay was used as described previously [12]. In brief, aliquots of whole blood collected from the trauma patients immediately after admission were mixed 1:1 with RPMI 1640 culture medium and incubated with 100 ng/ml lipopolysaccharide (LPS) (*Escherichia coli* O26:B6; Difco Laboratories, Detroit, MI, USA) in a sample mixer at 37 °C for 4 h. The supernatants were carefully collected after centrifugation and stored at −80 °C for assays of tumor necrosis factor (TNF)-α production, which was determined by enzyme-linked immunosorbent assay according to the manufacturer’s instructions (Endogen, Woburn, MA, USA). FPR2/ALX RNA and protein expression was detected using real-time quantitative polymerase chain reaction (qPCR) and flow cytometric analysis.

### Flow cytometric analysis

Flow cytometric analysis was used to detect the FPR2/ALX protein expression. Cells (1 × 10^6^ cells/ml) were incubated with 10 μl of fluorescein isothiocyanate (FITC)-conjugated antihuman FPR2/ALX monoclonal antibodies (Bio-Techne, Minneapolis, MN, USA) and Alexa Fluor 647-labeled secondary antibody (Invitrogen, Carlsbad, CA, USA). Analyses were carried out using an Accuri C6 flow cytometer (BD Biosciences, San Jose, CA, USA). Isotype-matched murine immunoglobulins (FITC-conjugated monoclonal immunoglobulin G2b [IgG2b] and phycoerythrin-conjugated monoclonal IgG2a antibody) with no reactivity to the antigen under study were used to adjust the negative fluorescence threshold.

### Plasmid construction and luciferase reporter assay

A 2167-bp promoter sequence (−2000 to +167) of the *FPR2/ALX* gene was created by PCR amplification of the genomic DNA, which was collected from a patient who was homozygous for the G allele at position −1010. The PCR primer sequences were as follows: 5′-CGGG*GTAC*CAGCAAAGACTTGGAACCAACCCAAATGTCCAACAA-3′ (forward) and 5′-GGAA*GATC*TGATAGAAACATAGGCACTCAAAAGCCACCTGTGGCA-3′ (reverse). The forward primer introduced a 5′ *Kpn*I restriction enzyme site, and the reverse primer introduced a 5′ *Bgl*II restriction enzyme site, respectively. The mutagenesis primer sequences were as follows: *FPR2*-2-F: 5′-CCTCCGGATATTGACTCTGGATCCGTGAATC-3′, *FPR2*-2-R: 5′-CAGAGTCAATATCCGGAGGAGGGAACATGTA-3′. Human embryonic kidney 293 cells were cotransfected with the constructed vectors or pGL3-basic original plasmid and 15 ng of control Renilla luciferase reporter plasmid pRL-cytomegalovirus using the Lipofectamine 2000 system (Invitrogen). The detailed vector construction method and luciferase reporter assay were described previously [12].

### RNA extraction and real-time qPCR

For qPCR of FPR2/ALX mRNA expression, total RNA was isolated from peripheral leukocytes, which were derived from whole blood collected from LPS-stimulated trauma patients using TRIzol reagent (Life Technologies, Carlsbad, CA, USA) according to the manufacturer’s protocol. β-Actin served as an internal control. The complementary DNAs (cDNAs) for real-time qPCR were synthesized using total RNAs from cell lysates. cDNA was synthesized with an oligo(dT) primer in a 20-μl reaction from 1 μg of total RNA using the PrimeScript^TM^ reverse transcription system (Takara Bio, Shiga, Japan) according to the manufacturer’s instructions. cDNA (1 μl) was then added to SYBR Green PCR Master Mix (Takara Bio) and subjected to PCR amplification using an iCycler system (CFX96; Bio-Rad Laboratories, Hercules, CA, USA). Relative expression was calculated using the 2^−ΔΔCt^ comparative cycle threshold method with values normalized to the reference gene β-actin.

### Statistical analysis

Sample size was calculated using Power and Sample Size Program software (http://biostat.mc.vanderbilt.edu/wiki/Main/PowerSampleSize). The desired power of our study was set at 70% with a significance level of 0.05 in a two-sided test. We chose the log-additive inheritance model, which is the most suitable model for polygenic diseases. All statistical analyses were performed using PASW Statistics version 18.0 software (SPSS, Inc., Chicago, IL, USA). The Hardy-Weinberg equilibrium of the genotype distribution was assessed using a χ^2^ goodness-of-fit test. Luciferase activities, FPR2/ALX protein expression, and cytokine production were tested using one-way analysis of variance. The associations of *FPR2/ALX* variants with major clinical features, including sepsis, were evaluated using the χ^2^ test. ORs with 95% CIs were calculated by logistical regression. Univariate analyses were performed to evaluate the associations between clinical characteristics and the rs11666254 polymorphism with sepsis. Multivariate logistic regression analysis was performed to adjust for possible confounders. The variables considered potential predictors for sepsis were selected from the literature and from the clinical experience of our research group [4, 32, 33]. These variables consisted of patient characteristics (sex, age, MODS, organ dysfunction), type and severity of injury (Injury Severity Score [ISS], injured body regions), types of pathogens, and source of infection. A *P* value <0.05 was considered to be statistically significant.

## Results

### Overall clinical characteristics of patients with major trauma

A total of 646 subjects were successfully genotyped and enrolled. Clinical characteristics of the patients in this study cohort are summarized in Table 2. The majority of patients (81%; 523 male/123 female) were male. Patients were severely injured (ISS 24.3 ± 6.7) and were mostly young (mean age 41.8 ± 11.8 years). Overall, 308 cases had multiple severe injuries. Gram-negative infections were 22.5% and 22.9% of the pathogens identified in these two study cohorts. Gram-positive infections made up 15.64% and 15.65% of identified pathogens in the two cohorts of patients. The most common pathogens identified in this study were *Staphylococcus aureus*, *Escherichia coli*, coagulase-negative staphylococci, *Klebsiella pneumoniae*, *Enterococcus* spp., *Acinetobacter baumannii*, *Pseudomonas aeruginosa*, and *Enterobacter cloacae*. The most common source of cultures was the respiratory tract (Additional file 2). In general, in approximately 50% of the patients in both cohorts, no pathogens were found to be the causative microorganism for sepsis. Of the 646 patients included in this study, 199 (30.8%) developed sepsis, and the morbidity rates were 32.4% in females (*n* = 40) and 30.4% in males (*n* = 159). The median time point for sepsis occurrence in the whole study cohort was 7 days (interquartile range 5.0–9.0 days). The demographic and baseline characteristics, as well as the clinical data, were not significantly different between the two cohorts of severe trauma patients.

**Table 2:** Overall clinical characteristics of patients with major trauma

Clinical characteristics	Total	Cohort 1 (*n* = 275)	Cohort 2 (*n* = 371)	*P* value
Age, years, mean ± SD	41.8 ± 11.8	42.3 ± 11.8	41.4 ± 12.0	0.362
Age range, years	16–65	16–65	16–65	
Males/females, *n*	523/123	222/53	301/70	0.919
ISS, mean ± SD	24.3 ± 6.7	24.6 ± 6.5	24.2 ± 6.8	0.437
≥ 16 to <25, *n*	421	174	247	0.383
≥ 25, *n*	225	101	124	
Severely injured body regions, *n*				
Head	29	14	15	0.996
Thorax	99	42	57	
Abdomen	119	51	68	
Extremities	91	35	56	
Multiple	308	133	175	
MOD score	4.4 ± 2.0	4.6 ± 1.8	4.4 ± 2.2	0.212
Organ dysfunction, *n* (%)				
None	261	109	152	0.959
One, *n*	177	76	101	
Two, *n*	126	56	70	
Three or more, *n*	82	34	48	
Sepsis, *n*	199	84	115	0.931
Source of infection, *n*				
Respiratory tract	293	122	171	
Primary bloodstream	143	60	83	
Urinary tract	103	42	61	
Catheter-associated	40	18	22	
Wound	40	19	21	
Other^a^	26	14	12	
Pathogens, *n*				
Negative cultures	337	143	194	0.238
Gram-negative	147	62	85	
Gram-positive	101	43	58	
Fungi	31	9	22	
Multiple infection	30	18	12	

*ISS* Injury Severity Score, *MOD* Multiple organ dysfunction

^a^Other included other sources of infection, such as soft tissue, bone, ascites, mucosa, cerebrospinal fluid, nonconfirmed sources

### Association of FPR2/ALX tag SNPs with major clinical features in trauma patients

The association of *FPR2/ALX* tag SNPs was evaluated with clinical features in 275 trauma patients (Table 3). The genotype distributions of the three tag SNPs did not deviate from Hardy-Weinberg equilibrium (*P* > 0.05) (Additional file 3). The SNP rs11666254 was significantly associated with the risk of sepsis in trauma patients. The morbidity rates of sepsis were significantly different among cases carrying AA, GA, and GG (44.9%, 30.6%, and 22.8%, respectively; *P* = 0.025). The patients carrying the A allele (GA + AA) had significantly higher morbidity rates of sepsis than patients carrying only the G allele (GG) (34.4% vs 22.8%; *P* = 0.049). Neither of the other two SNPs, rs17695052 or rs17695064, was significantly associated with sepsis in trauma patients (*P* > 0.05). No significant differences in age, sex, ISS, or MOD score were observed between different genotypes for all three tag SNPs (*P* > 0.05).

**Table 3:** Clinical relevance of the three tag single-nucleotide polymorphisms of the *FPR2/ALX* gene in 275 trauma patients

SNP	Genotype	Number	Age (years)	Sex (M/F)	ISS	Sepsis, *n* (%)	MOD score
rs11666254	GG	92	42.0 ± 12.2	73/19	24.5 ± 5.9	21 (22.8)^a^	4.6 ± 1.7
	GA	134	42.7 ± 11.7	113/21	24.8 ± 7.1	41 (30.6)^a^	4.4 ± 1.9
	AA	49	41.5 ± 11.1	36/13	24.3 ± 5.7	22 (44.9)^a^	4.7 ± 1.3
rs17695052	AA	226	41.9 ± 11.5	185/41	24.4 ± 6.4	67 (29.6)	4.6 ± 1.8
	AG	45	43.6 ± 13.3	33/12	25.5 ± 6.7	17 (37.8)	4.4 ± 1.7
	GG	0	0	0	0	0	0
rs17695064	CC	233	42.2 ± 11.8	187/46	24.6 ± 6.3	72 (30.9)	4.6 ± 1.8
	CT	38	42.0 ± 12.0	31/7	24.7 ± 7.6	12 (31.6)	4.3 ± 1.8
	TT	0	0	0	0	0	0

*ISS* Injury Severity Score, *MOD* Multiple organ dysfunction, *SNP* Single-nucleotide polymorphism

Age and ISS are given as the mean ± SD; MOD score is given as the mean ± SE.

^a^GG vs GA vs AA, *P* = 0.025; *P* = 0.049, GG vs GA + AA for dominant effect (variant homozygotes + heterozygotes vs wild homozygotes) by analysis of covariance

To further confirm the clinical association of the rs11666254 polymorphism with the development of sepsis, an additional 371 trauma patients were genotyped for the SNP rs11666254. As shown in Table 4, a pooled analysis of 646 trauma patients resulted in a morbidity rate of sepsis of 30.80% (199/646). The GG, GA, and AA carriers accounted for 22.8%, 31.6%, and 43.6% of all sepsis cases, respectively. Univariate analyses showed that ISS, pathogen type, and the rs11666254 polymorphism were associated with sepsis (*P* < 0.05). Other factors, such as the source of infection, injured region, sex, and age were not found to be significantly associated with sepsis. Before we determined the morbidity rate according to different alleles, the dependent value/outcome was the rate of sepsis per genotype. After adjustment for possible confounders, including ISS and types of pathogens, logistic regression analyses showed that patients with the rs11666254 polymorphism had a higher risk of developing sepsis (OR 1.728, 95% CI 1.336–2.235; *P* = 0.000). Patients with the GA or AA genotype had a significantly higher risk of developing sepsis than patients with the GG genotype (GA vs GG OR 1.806, 95% CI 1.176–2.773, *P* = 0.007; and AA vs GG OR 3.009, 95% CI 1.788–5.062, *P* = 0.000, respectively) (Table 5).

**Table 4:** Association study with septic and nonseptic individuals among 646 major trauma patients

Clinical characteristics	Number of patients or mean ± SD	Sepsis (*n* = 199)	Nonsepsis (*n* = 447)	X/t	*P* value
Age, years, mean ± SD	41.8 ± 11.8	40.8 ± 7.4	42.2 ± 11.6	−1.397	0.163
Age range, years	16–65	16–65	16–65		
Males/females, *n*	523/123	159/40	364/83	0.210	0.665
ISS, mean ± SD	24.3 ± 6.7	26.4 ± 7.4	23.4 ± 6.1	5.007	0.000
≥ 16 to <25, *n*	421	103	318	22.789	0.000
≥ 25, *n*	225	96	129		
Severely injured body regions, *n*					
Head	29	7	22	3.487	0.993
Thorax	99	28	71		
Abdomen	119	37	82		
Extremities	91	28	63		
Multiple	308	99	209		
Organ dysfunction, *n*					
None	261	81	180	1.328	0.725
One, *n*	177	53	124		
Two, *n*	126	43	83		
Three or above, *n*	82	22	60		
Source of infection, *n* (%)					
Respiratory tract	293	89	204		
Primary bloodstream	143	50	93		
Urinary tract	103	33	70		
Catheter-associated	40	10	30		
Wound	40	11	29		
Others	26	6	20		
Pathogens, *n* (%)					
Negative cultures	337	82	255	23.841	0.001
Gram-negative	147	52	95		
Gram-positive	101	40	61		
Fungi	31	8	23		
Multiple infections	30	17	13		
rs11666254, *n* (%)					
GG	219	50	169	15.599	0.000
GA	310	98	212		
AA	117	51	66		
GA + AA	427	149	278	9.883	0.002
GG + GA	529	148	381	10.956	0.001

*ISS* Injury Severity Score, *X/t* Chi-square value/t value

**Table 5:** Logistic regression analysis of the rs11666254 polymorphism with sepsis morbidity (*n* = 646)

Variables	β	SE	Wald statistic	Significance	OR	95% CI	
						Low	High
Sex	0.044	0.235	0.035	0.851	1.045	0.659	1.656
Age	−0.010	0.008	1.697	0.193	0.990	0.975	1.005
ISS	0.052	0.014	14.736	0.000	1.054	1.026	1.082
MODS	0.326	0.048	46.673	0.000	1.385	1.262	1.521
Pathogens	0.023	0.015	2.549	0.110	1.024	0.995	1.053
Injured body regions	−0.006	0.006	0.908	0.341	0.994	0.981	1.007
Source of infection	−0.056	0.067	0.712	0.399	0.945	0.829	1.078
Organ dysfunction	−0.028	0.089	0.101	0.751	0.972	0.817	1.157
rs11666254 (GG)			17.599	0.000	1		
rs11666254 (GA)	0.591	0.219	7.298	0.007	1.806	1.176	2.773
rs11666254 (AA)	1.101	0.265	17.217	0.000	3.009	1.788	5.062
Consent	−3.564	0.626	32.404	0.000	0.028		

*ISS* Injury Severity Score, *MODS* Multiple Organ Dysfunction Score

### Promoter polymorphism SNP rs11666254 inhibits the *FPR2/ALX* gene at both the transcriptional and translational levels

**Fig. 1:**
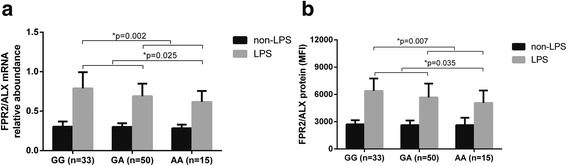
The single-nucleotide polymorphism rs11666254 inhibits lipopolysaccharide (LPS)-induced FPR2/ALX messenger RNA (mRNA) and protein expression. Data are presented as the mean and SD. The whole-blood samples collected from 98 trauma patients were mixed 1:1 (vol/vol) with RPMI 1640 culture medium and incubated with 100 ng/ml of *Escherichia coli* LPS (O26:B6) at 37 °C for 4 h. (**a**) *FPR2/ALX* mRNA and (**b**) protein expression in the peripheral leukocytes were assayed using quantitative polymerase chain reactions (presented as relative abundance) and flow cytometry (presented as mean fluorescence intensity [MFI]), respectively. One-way analysis of variance was used to assess statistical significance. **a** For mRNA expression, *P =* 0.002 for dominant association (GG vs GA + AA) and *P =* 0.025 for recessive effect (GG + GA vs AA). *P =* 0.012 for GG vs GA; *P =* 0.002 for GG vs AA; *P =* 0.151 for GA vs AA. **b** For protein expression (MFI), *P =* 0.007 for dominant association (GG vs GA + AA), and *P =* 0.035 for recessive effect (GG + GA vs AA). *P =* 0.029 for GG vs GA, *P =* 0.004 for GG vs AA, *P =* 0.163 for GA vs AA. There was no significant difference between genotypes before LPS stimulation

To determine whether the sequence variants of *FPR2/ALX* rs11666254 affected expression of the FPR2 mRNA and protein, we measured the FPR2 expression of peripheral leukocytes in response to ex vivo LPS stimulation in subjects with different genotypes. The LPS-inducible FPR2/ALX mRNA and protein expression was shown to be closely associated with the rs11666254 polymorphism. The A allele carriers had a significantly lower expression of FPR2/ALX mRNA. According to the results of the statistical analysis, there was a significant difference in both dominant and recessive effects (mRNA expression *P* = 0.002 for dominant effect and *P* = 0.025 for recessive effect; protein expression *P* = 0.007 for dominant effect and *P* = 0.035 for recessive effect) (Fig. 1 and Additional file 4). There was no significant difference in the total number of leukocytes between different genotype groups upon admission (GG 10.96 ± 5.36 × 10^9^ cells/ml, GA 11.55 ± 5.39 × 10^9^ cells/ml, AA 11.14 ± 5.21 × 10^9^ cells/ml, *P* > 0.05).

### rs11666254 is associated with LPS-induced TNF-α production

**Fig. 2:**
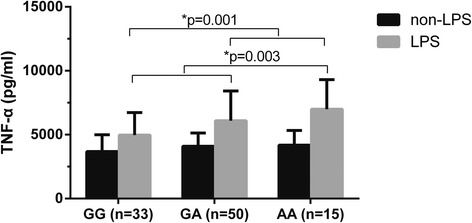
rs11666254 polymorphism and lipopolysaccharide (LPS)-induced tumor necrosis factor (TNF)-α production. The whole-blood samples collected from 98 trauma patients were treated as shown. TNF-α production was determined using a sandwich enzyme-linked immunosorbent assay. One-way analysis of variance was used to assess statistical significance. *P* = 0.001 for dominant association, GG vs GA + AA, *P* = 0.003 for recessive association. *P* = 0.029 for GG vs GA; *P* = 0.006 for GG vs AA; *P* = 0.187 for GA vs AA. There was no significant difference between genotypes before LPS stimulation

In animals with sepsis, *Fpr2/3* gene activation can be induced by TNF-α, and FPR2/ALX activation can decrease TNF-α levels [28]. Therefore, we hypothesized that functional variants of *FPR2/ALX* might be associated with TNF-α production. There was no statistically significant difference between these groups in the absence of LPS stimulation, but rs11666254 was found to be closely associated with higher LPS-induced TNF-α production. The TNF-α level was significantly higher in A allele carriers than in G allele carriers (*P* = 0.001 and 0.003 for dominant and recessive models, respectively) (Fig. 2).

### rs11666254 and transcription activity of *FPR2/ALX*

**Fig. 3:**
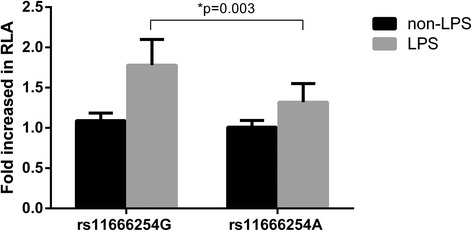
rs11666254 polymorphism and the transcription activity of the *FPR2/ALX* promoter. Relative luciferase activity (RLA) was used in human embryonic kidney cells transfected with rs11666254G or rs11666254A constructs as described in the Methods section. Luciferase activity was normalized for transfection efficiency using the control plasmid pRL-cytomegalovirus. These results are expressed as fold increases in RLA of the *FPR2/ALX* promoter construct vector compared with pGL3-basic (mean ± SD). *P = 0.03 vs the wild-type construct. *LPS* Lipopolysaccharide

Considering the location of the rs11666254 polymorphism in the 5′-flanking region of the *FPR2/ALX* gene, we hypothesized that the G-to-A variation of this location might affect the promoter activities of the *FPR2/ALX* gene. The luciferase reporter assay showed that the plasmid vector containing the A allele of rs11666254 had significantly lower transcription activity of *FPR2/ALX* than the vector containing the G allele with LPS induction (*P* = 0.003), but there was no significant difference in the transcription activity of *FPR2/ALX* between the two vectors without LPS induction (*P* > 0.05) (Fig. 3).

## Discussion

Trauma is one of the leading causes of death globally among young adults [1, 2]. Patients surviving the initial period after trauma are at risk of developing, and possibly dying as a result of, sepsis and sepsis-associated multiple organ dysfunction and failure. The disturbance of the regulation of inflammatory self-restriction is an important pathogenetic mechanism of sepsis [34, 35]. Proresolving mediators and their receptors (FPR2/ALX), which downregulate inflammation, are the brake signal molecules of inflammation. LXA4 was shown to inhibit polymorphonuclear neutrophil (PMN) migration, to induce chemotaxis in monocytes, and to promote the phagocytosis of apoptotic PMNs by macrophages [35].

In this study, we identify, for the first time to our knowledge, an SNP (rs11666254) located in the promoter of *FPR2/ALX* that is associated with increased sepsis hypersensitivity in major trauma patients. Our results show that rs11666254 was closely related to the sepsis morbidity rate after major trauma in a test cohort, and we confirmed the results in a validation trauma cohort. We then demonstrated its functional significance with respect to gene expression and ex vivo biological responses.

Our study shows that patients carrying the A allele of rs11666254 have higher risk of developing sepsis than G allele carriers. Between sepsis and nonsepsis individuals, we found that sex, ISS, pathogen type, and rs11666254 polymorphism were the important variables affecting sepsis morbidity; therefore, we used multiple logistic regression analysis to test for an independent effect of the rs11666254 polymorphism on the associations. Thus, our interpretation of the observed associations is less likely to be influenced by unmeasured confounders. Compared with patients who carried the GG genotype, patients carrying the GA or AA genotype had a significantly higher risk of developing sepsis (OR 1.81 and 3.01, respectively). Of the other two SNPs (rs17695052 and rs17695064) that were evaluated, we did not observe a significant association. No association was observed between the rs11666254 polymorphism and multiple organ dysfunction in sepsis patients. One of the reasons might be polygenetic and multifactorial involvement in the development of multiple organ dysfunction after trauma.

Researchers in a few studies have explored the asso ciation of *FPR2/ALX* polymorphisms with human disease. Gwinn et al. found that two SNPs (F110S and C126W), which were located in the open reading frame of *FPR2/ALX*, were associated with juvenile periodontitis [36]. Kim et al. observed that an intronic SNP (4209 T/G) was associated with the risk of asthma disease [37]. These three SNPs (F110S and C126W, 4209 T/G) along with 220A/G variants (data in the study by Simiele et al. [29]) were not analyzed in the present study. The reason is that in this study, the common SNPs in the *FPR2/ALX* gene with MAF greater than or equal to 0.05 were selected for the analysis of tSNPs, whereas the frequencies of these SNPs in the Chinese population is below 5%. A total of 21 SNPs in the *FPR2/ALX* gene from the HapMap database for the CHB population (Additional file 1) were enrolled. On the basis of analysis of SNP haplotypes in each block and tagging threshold of *r* ^2^, rs11666254 is the tag SNP in block 1 and rs17695052 is the tag SNP in block 2. rs17695064 was still selected because it is located in the 3′-UTR of exon 2, which might regulate *FPR2/ALX* gene transcription. Taken together, the three SNPs (rs11666254, rs17695052, and rs17695064) selected in this study for genotyping might capture most of the genetic variation of the entire *FPR2/ALX* gene and might represent potential biological significance of the *FPR2/ALX* genetic variations. Three SNPs in our study (rs11666254 [−1010G/A], rs7248161 [−1160G/G], and rs7256993 [398 T/T]) were not included in Kim et al.’s study [37]. The reason may be the relatively low MAF of the three SNPs in their study population.

*FPR2/ALX* is located on chromosome 19 [38]. Alternative splicing gives rise to four mRNAs in which there are different truncations at the 5′-UTR and different exon cassettes [29]. Along these lines, 15-epi-LXA4 biosynthesis and FPR2/ALX expression determine the magnitude and duration of the inflammatory reaction in humans [39]. In a previous study, a rare single-nucleotide mutation (A/G) located −220 bp upstream of the transcription start site was found to reduce the promoter activity as well as the mRNA and protein levels of FPR2/ALX [29].

How might the rs11666254 variant affect susceptibility to sepsis? To confirm the possible functional significance of the rs11666254 polymorphism, we further investigated the association of this polymorphism with *FPR2/ALX* expression using ex vivo stimulation of whole blood with LPS in trauma patients. The results show that the A minor allele was significantly and negatively associated with FPR2 expression, indicating that FPR2/ALX production in subjects with A carriers was significantly lower than in G carriers. Neutrophils are the primary source of soluble annexin A1 in inflammatory resolution [40]. Our finding that FPR2/ALX was highly expressed in PMNs (data not shown) supported the role of FPR2/ALX in signaling the critical step in resolution [41]. These results further validated the biological function of rs11666254, and we can conclude that rs11666254 not only was a useful biomarker for sepsis susceptibility posttrauma but also was a functional SNP affecting FPR2/ALX expression.

TNF-α is one of the most well-defined proinflammatory cytokines. Various evidence has shown that high serum TNF-α levels were positively correlated with the severity and prognosis of inflammatory diseases [42, 43]. Data derived from in vitro and animal experiment data have indicated that the *Fpr2/3* gene (an orthologue to human *FPR2/ALX*) is crucial to enacting nonredundant functions including control of cell recruitment, phagocytosis, modulation of soluble mediator generation, and containment of bacteremia, which prevents spread to vital organs and opens new opportunities to manipulate the host response in sepsis. The anti-inflammatory effect occurred mainly through the regulation of TNF-α [28]. The levels of FPR2/ALX expression might be an important determinant of LPS-induced TNF-α production [44]. Lipoxin and aspirin-triggered lipoxin inhibit TNF-α secretion from activated T cells via FPR2. Given the clinical relevance of the rs11666254 polymorphism, and on the basis of our results, we further hypothesized that this SNP might be associated with TNF-α production in patients with major trauma. An association was also observed between the rs11666254 A allele and higher TNF-α production. These results correlated with the clinical relevance of rs11666254.

This SNP is located −1010 bp upstream of the transcription start site of the *FPR2/ALX* gene. To further determine that the association of the rs11666254 polymorphism with *FPR2/ALX* production is due to the direct effect of this polymorphism rather than the effect of other polymorphisms in linkage disequilibrium with other polymorphisms, we investigated the effect of the rs11666254 polymorphism on the *FPR2/ALX* promoter activity using a reporter gene assay system. Our results showed that the fold increase of relative luciferase activity is significantly lower when transfected with vectors containing the rs11666254 A allele. The results suggest that G-to-A variation could significantly reduce the transcriptional activity of the *FPR2/ALX* promoter. However, the exact mechanism needs to be studied further.

Despite a sophisticated design, our study also has limitations. One limitation was that rare variants in the *FPR2/ALX* gene were not investigated. We estimated that our study had 89.9% power to detect a moderate association (OR 1.5) between a common variant (e.g., rs11666254) and sepsis. However, it had only 58.6% and 36.1% power to detect a moderate association (OR 1.5) for a rare variant with MAFs of 0.1 and 0.05, respectively. This result shows that larger samples are required to assess the association between rare variants of *FPR2/ALX* and sepsis. Another limitation was that the in vivo association of rs11666254 with *FPR2/ALX* expression and TNF-α level could not be confirmed in this study because of difficulties in obtaining blood samples from some sepsis patients.

In summary, the association between the common variants of *FPR2/ALX* and sepsis was evaluated in severe trauma patients. It was demonstrated that a promoter polymorphism in *FPR2/ALX* was capable of decreasing the level of gene transcription activity, downregulating mRNA and protein expression, and increasing TNF-α production with LPS induction. These results suggest that rs11666254 might increase the incidence of sepsis in patients with severe trauma. This polymorphism may be an important biomarker that can be used in the early risk assessment of sepsis after major trauma. However, the clinical application of this polymorphism has yet to be studied.

## Conclusions

The SNP rs11666254 in the promoter of *FPR2/ALX* increases sepsis susceptibility in patients following traumatic injury. Patients who carried the A allele of rs11666254 had a significantly higher risk of developing sepsis than individuals who carried the G allele. This SNP is associated with lower FPR2/ALX mRNA and protein expression in cells, and the A allele decreases the promoter activity of the *FPR2*/*ALX* gene.

## Additional files

Additional file 1: Table S1. Distribution of SNPs within the *FPR2/ALX* gene and in 3-kb regions upstream and downstream of the HapMap database for the CHB population. Tag SNPs were selected according to the HapMap CHB (Han Chinese in Beijing) data (version 3, release R2) using Haploview version 4.2. (DOCX 13 kb)

Additional file 2: Table S3. Summary of infection sources. We analyzed sources of infection in 646 major trauma patients. (DOCX 12 kb)

Additional file 3: Table S2. Distribution of the tSNPs of the *FPR2*/*ALX* gene in 275 trauma patients. We evaluated distribution of the three tSNPs of the *FPR2*/*ALX* gene in 275 trauma patients. (DOCX 12 kb)

Additional file 4: Figure S1. Flow cytometric analysis of FPR2 protein expression. FPR2 protein expression on the peripheral mononuclear cells and the polymorphonuclear cells. (DOCX 74 kb)

## Abbreviations

cDNA: Complementary DNA
CHB: Han Chinese in Beijing
FITC: Fluorescein isothiocyanate
FPR2/ALX: Formyl peptide receptor 2-lipoxin receptor
Ig: Immunoglobulin
ISS: Injury Severity Score
LPS: Lipopolysaccharide
LXA4: Lipoxin A4
MAF: Minor allele frequency
MFI: Mean fluorescence intensity
MOD: Multiple organ dysfunction score
MODS: Multiple organ dysfunction syndrome
mRNA: Messenger RNA
PMN: Polymorphonuclear neutrophil
qPCR: Quantitative polymerase chain reaction
RLA: Relative luciferase activity
SNP: Single-nucleotide polymorphism
TNF-α: Tumor necrosis factor-α
tSNP: Tag single-nucleotide polymorphism
UTR: Untranslated region
