# Supplementary material for: The common promoter polymorphism rs11666254 downregulates FPR2/ALX expression and increases risk of sepsis in patients with severe trauma
Source: Crit Care. 2017 Jul 6;21:171. doi: 10.1186/s13054-017-1757-3 (PMC5499024; doi:10.1186/s13054-017-1757-3)
Supplement: Supplementary file 1 — Distribution of SNPs within the FPR2/ALX gene and in 3-kb regions upstream and downstream of the HapMap database for the CHB population. Tag SNPs were selected according to the HapMap CHB (Han Chinese in Beijing) data (version 3, release R2) using Haploview version 4.2. (DOCX 13 kb) [file 13054_2017_1757_MOESM1_ESM.docx]

**Table S1. Distribution of SNPs within the *FPR2/ALX* gene and in 3-kb regions up and downstream of Hapmap database for the CHB population**

| No. | rs number | Location on Chr9 | Location on the gene | Variation | MAF | Region |
| --- | --- | --- | --- | --- | --- | --- |
| 1 | rs11666254 | 56954974 | -1010 | G/A | 0.341 | 5**′**flanking |
| 2 | rs7248161 | 56955104 | -1160 | G/G | 0 | 5**′**flanking |
| 3 | rs7256993 | 56956662 | 398 | T/T | 0 | Intron 1 |
| 4 | rs4801893 | 56957884 | 1620 | T/G | 0.478 | Intron 1 |
| 5 | rs13343463 | 56958187 | 1923 | T/T | 0 | Intron 1 |
| 6 | rs10410115 | 56958918 | 2654 | G/T | 0.061 | Intron 1 |
| 7 | rs10853843 | 56959373 | 3109 | A/C | 0.056 | Intron 1 |
| 8 | rs17694981 | 56959398 | 3134 | A/T | 0.1 | Intron 1 |
| 9 | rs17694990 | 56959515 | 3251 | T/G | 0.333 | Intron 1 |
| 10 | rs4801894 | 56960656 | 4392 | C/T | 0.056 | Intron 1 |
| 11 | rs4802863 | 56960784 | 4520 | C/T | 0.333 | Intron 1 |
| 12 | rs17834679 | 56961899 | 5635 | C/G | 0.367 | Intron 1 |
| 13 | rs17695020 | 56961974 | 5710 | T/C | 0.067 | Intron 1 |
| 14 | rs17756793 | 56962359 | 6095 | G/A | 0.067 | Intron 1 |
| 15 | rs17695032 | 56962754 | 6490 | T/C | 0.067 | Intron 1 |
| 16 | rs17756805 | 56962830 | 6566 | C/A | 0.067 | Intron 1 |
| 17 | rs12984050 | 56963121 | 6857 | A/A | 0 | Intron 1 |
| 18 | rs3764541 | 56963568 | 7304 | C/T | 0.211 | Intron 1 |
| 19 | rs17695052 | 56965038 | 8774 | A/G | 0.067 | exon 2 (3'UTR) |
| 20 | rs17695064 | 56965069 | 8805 | C/T | 0.057 | exon 2 (3'UTR) |
| 21 | rs7250333 | 56967935 | 11671 | A/A | 0 | 3'flanking |

Genetic variation data for the FPR2/ALX gene and 3kb up and downstream region was obtained from the Hapmap project (www. Hapmap.org) for 45 Chinese Han Beijing (CHB) population. MAF indicates minor allele frequency.
