# Supplementary material for: The common promoter polymorphism rs11666254 downregulates FPR2/ALX expression and increases risk of sepsis in patients with severe trauma
Source: Crit Care. 2017 Jul 6;21:171. doi: 10.1186/s13054-017-1757-3 (PMC5499024; doi:10.1186/s13054-017-1757-3)
Supplement: Supplementary file 2 — Summary of infection sources. We analyzed sources of infection in 646 major trauma patients. (DOCX 12 kb) [file 13054_2017_1757_MOESM2_ESM.docx]

| Sources of infection | N |
| --- | --- |
| Respiratory tract infection | **293** |
| Single respiratory tract infection | 110 |
| Respiratory tract combined bloodstream infection | 116 |
| Respiratory tract combined wound infection | 10 |
| Respiratory tract combined urinary tract infection | 21 |
| Respiratory tract combined catheter infection | 25 |
| Respiratory tract combined bloodstream and wound infection | 5 |
| Respiratory tract combined urinary tract and catheter infection | 6 |
| Primary bloodstream infection | **143** |
| Single bloodstream infection | 5 |
| Bloodstream combined respiratory tract infection | 116 |
| Bloodstream combined urinary tract infection | 18 |
| Catheter combined urinary tract infection | 4 |
| Urinary tract infection | **103** |
| Single urinary tract infection | 39 |
| Urinary tract combined respiratory tract infection | 27 |
| Urinary tract combined wound infection | 15 |
| Urinary tract combined bloodstream infection | 18 |
| Urinary tract combined cathether infection | 4 |
| Catheter-associated infection | **40** |
| Single catheter infection | 2 |
| Catheter combined respiratory infection | 31 |
| Catheter combined urinary tract infection | 4 |
| Catheter combined wound infection | 3 |
| Wound infection | **40** |
| Single wound infection | 7 |
| Wound combined urinary tract infection | 15 |
| Wound combined catheter infection | 3 |
| Wound combined respiratory tract infection | 15 |
| Other (soft tissue, bone, ascites, mucosa, cerebrospinal fluid, etc.) | **26** |

**Table S3. Summary of sources of infection**
