# Supplementary material for: The common promoter polymorphism rs11666254 downregulates FPR2/ALX expression and increases risk of sepsis in patients with severe trauma
Source: Crit Care. 2017 Jul 6;21:171. doi: 10.1186/s13054-017-1757-3 (PMC5499024; doi:10.1186/s13054-017-1757-3)
Supplement: Supplementary file 3 — Distribution of the tSNPs of the FPR2/ALX gene in 275 trauma patients. We evaluated distribution of the three tSNPs of the FPR2/ALX gene in 275 trauma patients. (DOCX 12 kb) [file 13054_2017_1757_MOESM3_ESM.docx]

**Table S2. Distribution of the tSNPs of the FPR2/ALX gene in 275 trauma patients**

| tag SNP | Region | N | MAF (%) | | | Genotypes, n (%) | | | HWE |
| --- | --- | --- | --- | --- | --- | --- | --- | --- | --- |
|  |  |  | Patients | Databank* | | Wildtype | Heterozygous | Variant |  |
| rs11666254 | 5**′**flanking | 275 | 39.6 | | 34.1 | 92 (33.5) | 134 (48.4) | 49 (17.7) | 0.10 |
| rs17695052 | 3**′**-UTR | 271 | 8.5 | | 6.7 | 226 (83.4) | 45 (16.6) | 0 (0) | 0.12 |
| rs17695064 | 3**′**-UTR | 271 | 8.0 | | 5.7 | 233 (86.0) | 38 (14.0) | 0 (0) | 0.14 |

*Data was from HapMap database for Chinese Han Beijing residents. MAF: minor allele frequency, HWE: Hardy-Weinberg equilibrium
