# Supplementary material for: The common promoter polymorphism rs11666254 downregulates FPR2/ALX expression and increases risk of sepsis in patients with severe trauma
Source: Crit Care. 2017 Jul 6;21:171. doi: 10.1186/s13054-017-1757-3 (PMC5499024; doi:10.1186/s13054-017-1757-3)
Supplement: Supplementary file 4 — Flow cytometric analysis of FPR2 protein expression. FPR2 protein expression on the peripheral mononuclear cells and the polymorphonuclear cells. (DOCX 74 kb) [file 13054_2017_1757_MOESM4_ESM.docx]

Figure S1 Flow cytometry analysis of FPR2 protein expression (A) on the peripheral mononuclear cells (B) on the polymorphonuclear (PMN) cells (presented as mean fluorescence intensity (MFI)). Mononuclear cells or polymorphonuclear cells were gated by forward scatter/side scatter.
